# *ETS1* variants confer susceptibility to ankylosing spondylitis in Han Chinese

**DOI:** 10.1186/ar4530

**Published:** 2014-04-04

**Authors:** Shan Shan, Jie Dang, Jiangxia Li, Ze Yang, Hailing Zhao, Qian Xin, Xiaochun Ma, Yongchao Liu, Xianli Bian, Yaoqin Gong, Qiji Liu

**Affiliations:** 1 Key Laboratory for Experimental Teratology of the Ministry of Education and Department of Medical Genetics, Shandong University School of Medicine, Jinan, Shandong 250012, China; 2 Department of Medical Genetics and Cell Biology, Ningxia Medical University, Yinchuan, Ningxia 750004, China; 3 Institute of Geriatrics, Beijing Hospital, Ministry of Health of PR China, Beijing 100730, China

## Abstract

**Introduction:**

*ETS1* is a negative regulator of the Th17 differentiation gene and plays a central role in the pathogenesis of autoimmune diseases. We aimed to investigate whether polymorphisms in *ETS1* confer susceptibility to ankylosing spondylitis (AS) in Han Chinese.

**Methods:**

We selected seven single nucleotide polymorphisms (SNPs) within *ETS1* based on HapMap data and previous genome-wide association study. Genotyping involved the TaqMan method in 1,015 patients with AS and 1,132 healthy controls from Shandong Province, and 352 AS patients and 400 healthy controls from Ningxia, a northwest region in China. Gene expression was determined by real-time PCR.

**Results:**

The SNP rs1128334 was strongly associated with AS (odds ratio 1.204, 95% confidence interval 1.06-1.37; *P* = 0.005). This association was confiexrmed in the Ningxia population (*P* = 0.015). Carriers of the haplotype TAT for rs12574073, rs1128334 and rs4937333 were associated with increased risk of AS and haplotype CGC with reduced risk as compared to controls. In addition, *ETS1* expression was lower in AS patients than controls. The risk allele A of rs1128334 and haplotype A-T of rs1128334 and rs4937333 were associated with decreased expression of *ETS1*.

**Conclusions:**

Common variants in *ETS1* may contribute to AS susceptibility in Han Chinese people.

## Introduction

Ankylosing spondylitis (AS) is a common inflammatory arthritis that affects the axial skeleton and peripheral joints [1]; it is usually accompanied by lower back pain, peripheral arthritis, enthesis and iritis, and even spinal deformity and ankylosis, ultimately limiting the mobility of the spine and other joints [2]. Genetic factors are strongly implicated in the pathogenesis of the disease, and the estimated heritability is up to 97% according to a twin study [3,4].

The major histocompatibility complex (MHC), mostly from human leukocyte antigen B27 (HLA-B27), accounts for nearly half of the predisposition for AS [5]. Although HLA-B27 is strongly associated with risk of AS, its associated genes account for only 20% to 30% of the overall genetic risk of AS [1]. Thus, other loci may be involved. Besides HLA-B27, non-MHC genes implicated in the risk of AS include *ERAP1, KIF21B, IL23R* and two intergenic regions at chromosome 2p15 and chromosome 21q22 in a European population [6,7].

Two recent genome-wide association studies (GWASs) of an Asian population demonstrated a novel region containing *ETS1* at chromosome 11q23 strongly associated with systemic lupus erythematosus (SLE) [8,9]. rs6590330, rs1128334 and rs4937333 were identified as the susceptible variants associated with SLE. Subsequently, many genetic studies reported that rs11221332 of *ETS1* is associated with rheumatoid arthritis (RA) and celiac disease, both characterized by excessive activation of the immune system [10,11]. Also, increasing evidence has suggested that many of the known AS-associated loci overlap with those of other immune-related diseases. Because of clinical and immunological overlap of AS and other autoimmune diseases [12], *ETS1* may be associated with AS as well. In this study, we investigated the association of *ETS1* genetic polymorphisms and AS in Han Chinese people.

## Methods

### Subjects

We recruited 2,899 unrelated subjects in this case-control study from 2008 to 2011, including 1,015 patients (701 male, 69.1%) with AS from Shandong Provincial Hospital and Qilu Hospital in Jinan, Shandong province. All patients, at a mean age of 38 ± 8.3 years, had no history of inflammatory bowel disease and/or psoriasis and met the modified New York criteria for AS. We also recruited 1,132 unrelated, randomly sampled healthy controls (603 male, 53.3%) from a health check-up center at the hospital during the same period; the mean age was 40 ± 7.2 years old. All subjects were ethnic Han living in Shandong, an eastern coastal province of North China. More detailed information about the samples was reported previously [13]. The replication samples of 352 AS patients and 400 controls from Ningxia (a province in northwest China) were provided by Professor Yang Ze, with detailed information [14]. Flow cytometry was used to immunophenotype HLA-B27 in patients and controls, and only HLA-B27-positive patients and HLA-B27-negative controls were recruited.

Genomic DNA from peripheral blood leukocytes was extracted by a standard phenol-chloroform method. The study was approved by the ethics committee for human studies at the Shandong University School of Medicine, and all subjects provided their informed consent.

### Sequence analysis of the human *ETS1* gene

All nine coding exons and 1,000 bp of the 5′ upstream sequence of *ETS1* were sequenced in DNA samples from 100 AS cases to screen for genetic variants. *ETS1* was amplified by PCR before sequencing. The PCR reaction was run in a total volume of 50 μL solution containing 5 × PCR buffer, 10 μL; 5 × dNTP (1 mmol/L each of dATP, dTTP, dGTP and dCTP), 10 μL; 50 ng genomic DNA; 0.4 μM of each primer; and 1 U Taq DNA polymerase. The PCR amplification conditions were optimized with an initial denaturation step at 94°C for 5 minutes followed by 35 cycles of 94°C for 40 sec, optimal annealing temperature for 40 sec, extension at 72°C for 40 sec and a final extension step of 72°C for 10 minutes. All sequencing was performed by the Genomic Analysis Facility (Shenzhen Huada, Shenzhen, China) and analyzed with use of Chromas 2.33 software.

### Single nucleotide polymorphism (SNP) selection and genotyping

**Figure 1:**
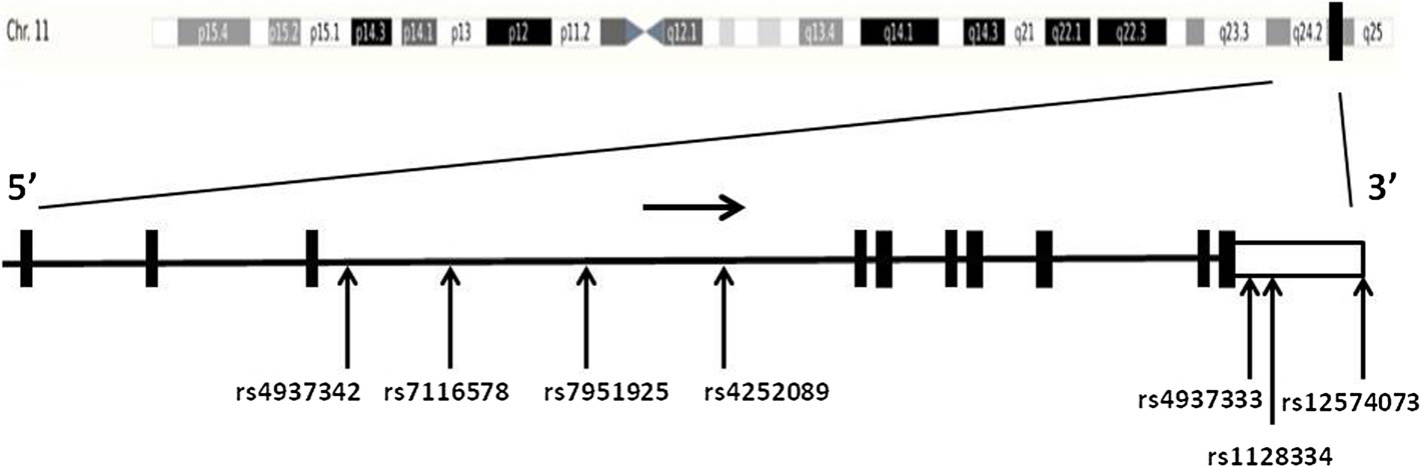
**Schematic location of seven single nucleotide polymorphisms (SNPs) in the *ETS1 *** **gene.**

Because we were unable to detect novel variants in the sequencing region, we selected seven SNPs (rs12574073, rs1128334, rs4937333, rs4254089, rs7951925, rs7116578 and rs4937342) in *ETS1* with minor allele frequency ≥0.05 and *r*^2^ >0.8 in data for Han Chinese in Beijing (HCB) in HapMap [15] and genotyped by TaqMan SNP genotyping assay using assay-on-demand probes and primers (C_31697960_10 for rs12574073, C_7539918_10 for rs1128224, C_278298_10 for rs4937333, C_449693_10 for rs4254089 C_220576_10 for rs7951925, C_29363433_10 for rs7116578, C_27979762_20 for rs4937342; Applied Biosystems, Foster City, CA, USA). rs1128334 and rs4937333 were reported to be strongly associated with SLE and were therefore selected and investigated. We also selected rs12574073 as a candidate SNP because it was a proxy SNP for rs6590330, demonstrated to be associated with SLE in a previous GWAS [9]. The locations of these SNPs are shown in Figure 1. All genotyping involved use of the Lightcycler 480 real-time PCR system (Roche, Mannheim, Germany). The PCR reaction was run in a total volume of 5 μL solution containing 2 × Premix EX Taq (Takara, Otsu, Shiga, Japan), 2.5 μL; >10 ng genomic DNA; 2.5 μM of each primer; and 0.05 μL probes. The PCR amplification conditions were 95°C for 30 sec, 45 cycles of 95°C for 5 sec, 60°C for 20 sec and 40°C for 30 sec for cooling. Genotyping accuracy in the samples was confirmed by direct sequencing of PCR products for 5% of randomly chosen samples.

### RT-PCR

Total peripheral leukocyte RNA was extracted from 50 AS patients and 161 healthy controls by the TRIzol reagent method (Invitrogen, Carlsbad, CA, USA), then underwent reverse transcription by use of a reverse transcript kit (Takara, Otsu, Shiga, Japan). Allelic-specific expression of *ETS1* was analyzed only in healthy controls. RNA samples were treated with RNase free DNAase to eliminate genomic DNA contamination before being reverse-transcribed into cDNA. Real-time quantitative RT-PCR to amplify cDNA involved the Roche 480 real-time PCR system with SYBR Green Master Mix (Applied Biosystems, Foster City, CA, USA). Relative expression was analyzed by the comparative threshold cycle (Ct) method and normalized to that of human glyceraldehyde-3-phosphate dehydrogenase (GAPDH), calculated by the 2^-△△Ct^ method and log^10^ transformed. The primer sequences for *ETS1* were sense 5′-TACACAGGCAGTGGACCAATC-3′ and antisense 5′-CCCCGCTGTCTTGTGGATG-3′; and GAPDH, sense 5′-CCAGGTGGTCTCCTCTGACTT-3′ and antisense 5′-GTTGCTGTAGCCAAATTCGTTGT-3′. Melting curve analysis was used to confirm specificity, with four duplicate wells used for each subject [16].

### Statistical analysis

The SNPs were tested for adherence to Hardy-Weinberg equilibrium (HWE) by chi-square test. Comparison of the allele frequency of the seven SNPs for patients and controls involved Fisher’s exact test with use of Plink 1.07 [17]. The Bonferroni correction was used for multiple test correction. Pairwise linkage disequilibrium (LD) was measured with use of Haploview 4.2. Three-locus haplotypes were calculated by use of a full precise-iteration algorithm with the online SHEsis software [18]. Differences in allelic expression were tested by *t-*test and analysis of variance (ANOVA) by use of GraphPad Prism v5.01 (GraphPad Software, La Jolla, CA, USA). *P* <0.05 was considered statistically significant.

## Results

### Sequencing

To search for new variants in the *ETS1* gene, we sequenced all exons including the untranslated region (UTR) and 5′ upstream regulatory region. However, no novel variant was found in any of the nine exons or 1,000 bp of the upstream sequence.

### Association study

We obtained allele, genotype and haplotype distributions for seven markers in *ETS1* in chromosome 11q23 for 1,132 controls and 1,015 patients with AS in Shandong. The allele frequency for each SNP was analyzed (Table 1), and none deviated significantly from HWE, as determined at the 0.05 significance level. Frequency of the rs1128334 allele A was significantly higher in AS patients (*P* = 0.005, odds ratio (OR) 1.204, 95% CI 1.06, 1.37) than in healthy controls. Even after the Bonferroni correction, the association remained significant (*Pc* = 0.035). rs12574073 and rs4937333 were associated with AS but not after the Bonferroni correction. Then, we replicated the associated SNP rs1128334 in the Ningxia cohort of 352 cases and 400 matched controls. The minor allele frequency of rs1128334 was greater for cases than controls (*P* = 0.015, OR 1.31, 95% CI 1.06, 1.62).

**Table 1:** Genotype and allele association analysis of seven single nucleotide polymorphisms (SNPs) in samples of AS in Shandong and rs1128334 in Ningxia

**Population**	**SNPs**	**Position**	**Genotype/allele**	**Patients, number (%)**	**Controls, number (%)**	**Odds ratio**	**95% CI**	***P-*** **value**	P*
	rs12574073	128319478	CC	423 (42.7)	401 (47.8)	1			
			CT	457 (46.1)	356 (41.8)	1.217	1.002, 1.478	0.192	
			TT	111 (11.2)	89 (10.4)	1.182	0.867, 1.613		
			C	1,303 (65.7)	1,170 (68.7)	1			
			T	679 (34.3)	534 (31.3)	1.142	0.994, 1.311	0.062	
	rs1128334	128328959	GG	417 (42.2)	528 (48.0)	1			
			AG	457 (46.3)	471 (42.8)	1.229	1.024, 1.473	0.018	
			AA	114 (11.5)	101 (9.2)	1.429	1.062, 1.923		
			G	1,291 (65.3)	1,527 (69.4)	1			
			A	685 (34.7)	673 (30.6)	1.204	1.058, 1.371	0.005	0.035
	rs4937333	128330520	TT	183 (18.2)	177 (15.6)	1			
			CT	483 (48.0)	535 (42.3)	0.873	0.687, 1.111	0.160	
			CC	341 (33.8)	420 (37.1)	0.785	0.611, 1.010		
			T	849 (42.2)	889 (39.3)	1			
			C	1165 (57.8)	1375 (60.7)	0.887	0.785, 1.003	0.057	
Shandong	rs4254089	128366221	CC	64 (6.3)	66 (5.9)	1			
			CT	356 (35.1)	365 (32.9)	1.006	0.692, 1.461	0.478	
			TT	595 (58.6)	680 (61.2)	0.902	0.629, 1.295		
			C	484 (23.8)	497 (22.4)	1			
			T	1546 (76.2)	1725 (77.6)	0.920	0.798, 1.062	0.259	
	rs7951925	128379964	AA	84 (8.3)	109 (9.7)	1			
			AG	420 (41.4)	444 (39.6)	1.227	0.896, 1.681	0.433	
			GG	510 (50.3)	569 (51.7)	1.163	0.854, 1.584		
			A	588 (29.0)	662 (29.5)	1			
			G	1,440 (71.0)	1,582 (70.5)	1.025	0.898, 1.169	0.736	
	rs7116578	128388773	AA	869 (85.9)	970 (86.3)	1			
			AG	133 (13.1)	145 (12.9)	1.024	0.795, 1.318	0.884	
			GG	10 (1.0)	9 (0.8)	1.240	0.502, 3.066		
			A	1,871 (92.4)	2,085 (92.7)	1			
			G	153 (7.6)	163 (7.3)	1.046	0.832, 1.316	0.726	
	rs4937342	128398309	TT	903 (89.8)	993 (90.2)	1			
			TG	101 (10.0)	102 (9.3)	1.089	0.815, 1.455	0.368	
			GG	2 (0.2)	6 (0.5)	0.367	0.074, 1.821		
			T	1,907 (94.8)	2,088 (94.8)	1			
			G	105 (5.2)	114 (5.2)	1.008	0.768, 1.324	1.000	
Ningxia	rs1128334	128328959	GG	135 (38.4)	190 (47.5)	1			
			AG	168 (47.7)	166 (41.5)	1.424	1.047, 1.938	0.038	
			AA	49 (13.9)	44 (11.0)	1.576	0.986, 2.490		
			G	438 (62.2)	546 (68.2)	1			
			A	266 (37.8)	254 (31.8)	1.305	1.055, 1.615	0.015	

*Adjusted *P*-value after Bonferroni correction.

### LD evaluation and haplotype analysis

**Figure 2:**
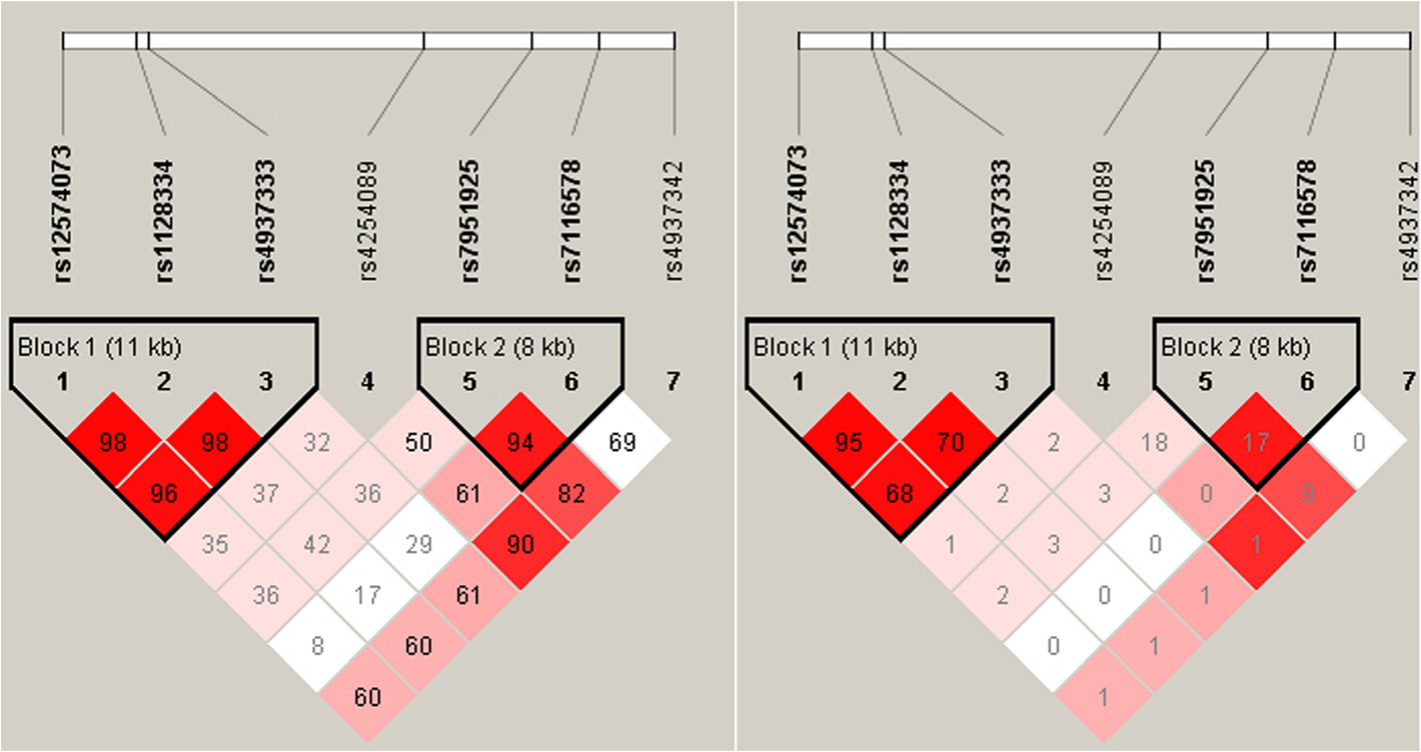
**Linkage disequilibrium of seven single nucleotide polymorphisms (SNPs) in the *ETS1 *gene.** Left, D’. Right, *r*^2^.

rs1128334 was in strong LD with rs12574073 (*r*^2^ = 0.95) (Figure 2). We identified eight different haplotypes in the studied population, but only three showed frequency >3% (Table 2). As expected, the haplotype results were similar to the single-site association results, and haplotype TAT formed by rs12574073, rs1128334 and rs4937333 was strongly associated with increased risk of AS (OR 1.24, 95% CI 1.05, 1.47, *P* = 0.01), whereas haplotype CGC reduced the risk (OR 0.82, 95% CI 0.70, 0.96, *P* = 0.01).

**Table 2:** Haplotype frequencies of three single nucleotide polymorphisms in all samples

**Haplotype**	**No. (%) of patients**	**No. (%) of controls**	**Odds ratio (95% CI)**	***P-*** **value**
T A T	478.82 (0.334)	345.87 (0.288)	1.24 (1.05, 1.47)	0.01
C G C	825.82 (0.577)	749.74 (0.624)	0.82 (0.70, 0.96)	0.01
C G T	112.17 (0.078)	93.24 (0.078)	1.01 (0.76, 1.35)	0.94

The order of three SNPs: rs12574073, rs1128334, rs4937333.

### mRNA expression of *ETS1* in peripheral blood mononuclear cells (PBMCs)

**Figure 3:**
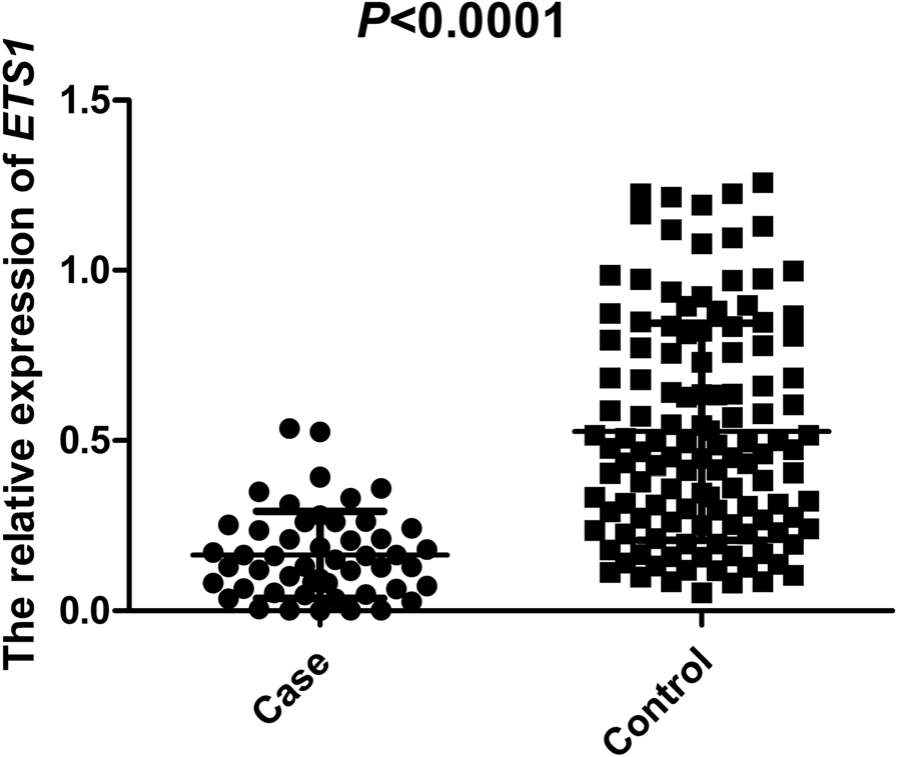
**mRNA expression of *ETS1 *** **in patients and controls.**

We examined the mRNA levels of *ETS1* in 50 AS patients and 161 healthy controls. Relative *ETS1* mRNA expression was lower in cases than controls (*P* <0.0001, Figure 3).

### Allelic-specific expression of *ETS1*

**Figure 4:**
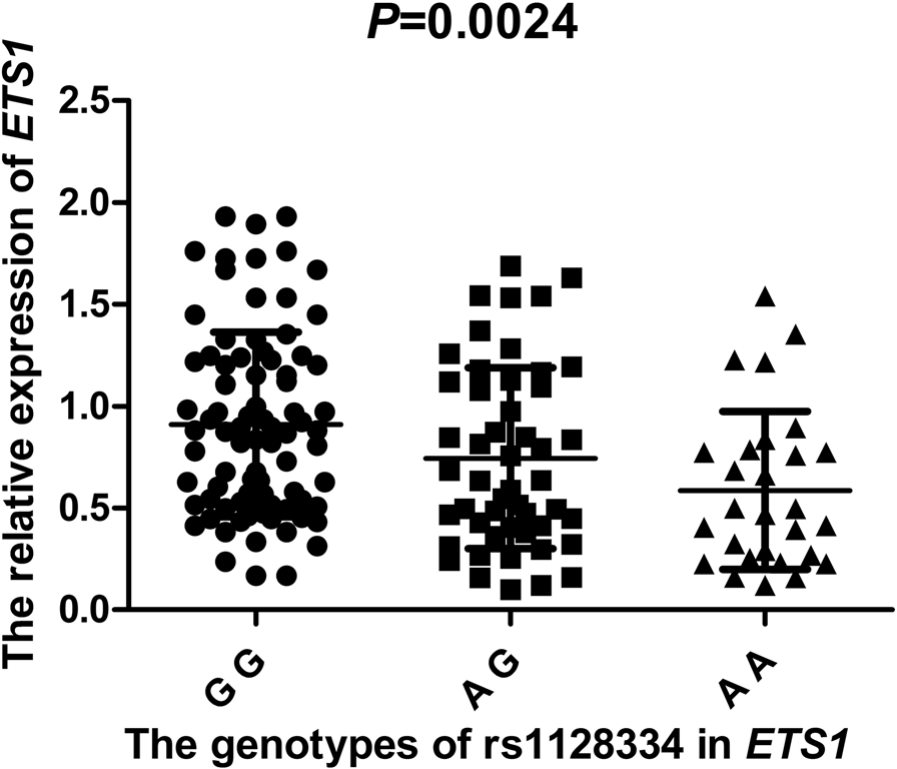
**Allelic-specific expression of *ETS1 *** **by genotypes of rs1128334.**

**Figure 5:**
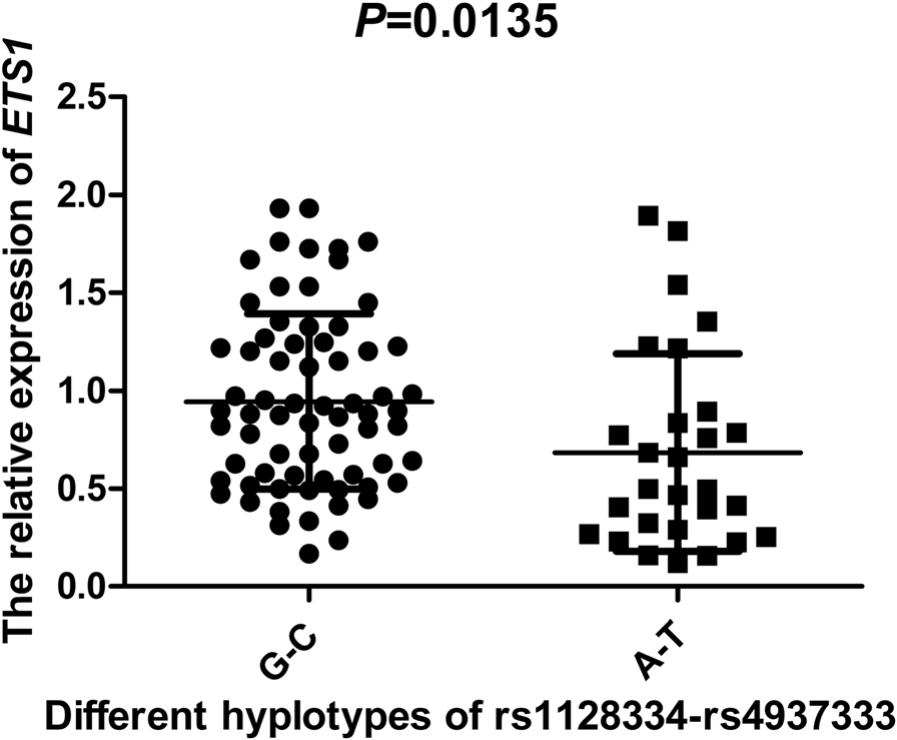
***ETS1 *** **mRNA levels in the carriers of each rs1128334-rs4937333 haplotype.**

To determine whether rs1128334 was associated with *ETS1* expression, we genotyped 161 healthy control samples for rs1128334 to compare the *ETS1* expression in subjects with different genotypes of the SNP. *ETS1* mRNA level was lower in AA (n = 28) and AG (n = 50) than GG (n = 83) homozygotes (*P* <0.01, Figure 4). Thus, carriers with the risk allele A of rs1128334 may exhibit significantly lower *ETS1* mRNA expression than those with the allele G. In addition, analysis of healthy controls involved only rs1128334 and rs4937333 because of the high LD between rs12574073 and rs1128334 (*r*^2^ = 0.95, Figure 2). Subjects carrying haplotype A-T (n = 28) for rs1128334/rs4937333 had a significantly lower level of *ETS1* than those carrying haplotype GC (n = 69) (*P* <0.05, Figure 5).

## Discussion

We have demonstrated that *ETS1* is associated with AS, thus adding to the list of loci showing overlap between AS, RA and SLE. We identified an SNP, rs1128334, located in the 3′ UTR of *ETS1*, which was significantly associated with AS in Han Chinese people. We also showed a lower expression of *ETS1* in peripheral leukocytes from patients than controls. Furthermore, the risk allele of rs1128334 and the risk haplotype A-T of rs1128334 and rs4937333 were significantly linked to decreased mRNA level of *ETS1*. To our knowledge, this is the first report demonstrating an association of *ETS1* polymorphisms and AS in Han Chinese. Especially, we verified the association of rs1128334 and AS in two populations, Han Chinese from Shandong and Ningxia, which enhances the credibility of our findings.

In previous reports, the common variants rs1128334, rs4937333 and rs6590330 were found to be significantly associated with SLE in east Asian populations. In addition, rs11221332 was suggested to be strongly associated with celiac disease in a European population and RA in a Caucasian population. In agreement with these studies of autoimmune diseases, we observed that *ETS1* was also a susceptibility gene for AS at least in northern Han Chinese. Our findings provide support for many autoimmune diseases sharing the same susceptibility loci.

*ETS1* encodes a member of the ETS family of transcription factors that activate transcription by binding to cis-regulatory elements in target genes [19,20]. The *ETS1* transcription factor was initially discovered as the proto-oncogene corresponding to v-ets of the avian erythroblastosis virus (E26) [19,21], which harbors a conserved DNA-binding domain mediating specific DNA binding to the GGAA/T motif [20]. As a crucial transcription factor widely expressed in lymphocytes and vascular endothelial, lacteal glandular epithelium and various invasion tumor cells, *ETS1* regulates the development, senescence and death of many immune cells [22,23]. It also plays a role in both innate and adaptive immune response [23,24]. Accumulating evidence points to an important role for *ETS1* in regulating the differentiation of immune cells such as T-cell differentiation into a helper population, terminal differentiation of B cells, development of natural killer (NK) cells and NK T cells and the expression of cytokine and chemokine genes in a wide variety of different cell lineages [24-27]. Animal experiments showed that autoimmune disease developed in *ETS1*-knockout mice, as investigated by the production of high titers of autoantibodies, and immune cell infiltration into organs accounted for aberrations in lymphocyte differentiation [24].

In addition to roles in controlling the production of interferon-gamma in T helper (Th)1 cells [28] and driving normal Th2 cytokine production, *ETS1* is also involved in the Th17 cell lineage as a negative regulator of Th17 cell differentiation [29]. Increasing studies suggest that Th17 cells are involved in the pathogenesis of AS [30]. Recently, Zhang *et al*. examined serum IL-17 levels from 283 SLE cases and observed a significant synergistic epistatic interaction between two risk variants, rs10893872 and rs1128334, in the *ETS1* gene, and the haplotype formed by these variants was significantly associated with serum IL-17 levels in SLE patients [31], which supports a role for genetic interaction contributing to the complexity of autoimmune disease [31-33]. From our findings, subjects with AS carrying the risk allele A have significantly lower *ETS1* expression than those with the G allele. Similar to previous studies, the haplotype formed by rs12574073, rs1128334 and rs4937333 in *ETS1* was significantly associated with AS susceptibility. As well, individuals carrying haplotype AT for rs1128334/rs4937333 showed less *ETS1* expression than those carrying the GC haplotype. With the HCB data in HapMap, rs10893872 and rs4937333 are in perfect LD (*r*^2^ = 1), so our results are consistent with the Zhang *et al*. study.

Much evidence has confirmed that microRNAs (miRNAs) can regulate gene expression by sequence-specific binding to target mRNAs, but the binding affinity can be affected by SNPs residing in miRNA target sites, which may in turn affect the ability of miRNA to inhibit the mRNA translation into proteins or lead to degradation of the mRNA [34]. Indeed, recent research has demonstrated that SNPs at miRNA binding sites likely affect the expression of the miRNA target genes and thus, may contribute to susceptibility to autoimmune diseases [35]. Therefore, we presumed that variants rs1128334 and rs4937333 located in the 3′ UTR of *ETS1* may affect miRNA binding affinity and further influence the expression of the target gene, which may contribute to susceptibility to AS. However, the detailed mechanism of this functional relationship requires further investigation.

## Conclusions

In summary, our study identified that the common variants of *ETS1* confer susceptibility to AS. This study might provide further evidence to improve our understanding of the exact function of *ETS1* in the pathogenesis of autoimmune diseases.

## Abbreviations

ANOVA: analysis of variance; AS: ankylosing spondylitis; bp: base pairs; Ct: threshold cycle; ERAP1: Endoplasmic reticulum aminopeptidase 1; ETS1: v-ets avian erythroblastosis virus E26 oncogene homolog 1; GAPDH: glyceraldehydes-3-phosphate dehydrogenase; GWAS: genome-wide association study; CB: Han Chinese in Beijing; HLA: human leukocyte antigen; HWE: Hardy-Weinberg equilibrium; IL-17: interleukin 17; IL23R: interleukin 23 receptor; KIF21B: Kinesin family member 21B; LD: linkage disequilibrium; MHC: major histocompatibility complex; miRNA: microRNA; NK cell: natural killer cell; OR: odds ratio; PBMC: peripheral blood mononuclear cell; RA: theumatoid arthritis; RT-PCR: reverse transcription PCR; SLE: systemic lupus erythematosus; Th cell: T helper cell; UTR: untranslated region.

## Competing interests

The authors declare that they have no competing interests.

## Authors’ contributions

SS: data collection and analysis, manuscript writing and final approval of the manuscript. JD: conception and design, critical revision and final approval of the manuscript. JL: conception and design, manuscript writing and final approval of the manuscript. ZY: data collection and analysis, critical revision and final approval of the manuscript. HZ: data collection and analysis, critical revision and final approval of the manuscript. QX: data collection and analysis, critical revision and final approval of the manuscript. XM: conception and design, critical revision and final approval of manuscript. YL: data collection and analysis, manuscript writing and final approval of the manuscript. XB: data collection and analysis, critical revision and final approval of the manuscript. YG: conception and design, critical revision and final approval of the manuscript. QL: conception and design, data collection and analysis, manuscript writing, final approval of the manuscript. All authors read and approved the final manuscript.
